# Strategy integration, sustainable drivers of firm internationalization performance—Moderated by environmental uncertainty and firm capabilities

**DOI:** 10.1007/s10843-023-00328-3

**Published:** 2023-05-05

**Authors:** Bimbo Onaolapo Adejare, Ekpenyong Ekpenyong Udofia, Gbemi Oladipo Olaore

**Affiliations:** 1 Department of Business Administration, University of Minho, Braga, Portugal; 2 Department of Business Administration, University of Lagos, Lagos, Nigeria; 3 Department of Business Administration, Karabuk University, Karabuk, Turkey

**Keywords:** Nonmarket strategy, Strategy Integration, Customer satisfaction, Market and competition, Firm capabilities, Environmental uncertainty, Stratégie non marchande, Intégration stratégique, Satisfaction du clientèle, Marché et concurrence, Capacités de l'entreprise, Incertitude environnementale

## Abstract

The global environmental uncertainty and the need for an organization to maximize profit and satisfy the interest of wider nonmarket groups/stakeholders in the host market propel and reinforce the need for strategic integration to achieve sustainable internationalization performance. The interest of this article is to examine the relative impact of market, nonmarket strategy, and strategy integration on the performance of medium and large organizations in Portugal. Furthermore, environmental uncertainty and firm capabilities were used as moderation to evaluate the performance implication of these strategy configurations on firm market advantage in the host country. Given the need to examine the relationship between the latent and measured variables in this study, structural equation models were used to test the stated hypotheses, while confirmatory factor analyses were used to assess the fitness of our model. Our findings revealed that strategy integration provides a more sustainable competitive performance than either market or nonmarket strategy when used separately, especially in highly regulated and standardized business contexts such as Portugal. Furthermore, our findings show that organization needs to design market-oriented strategies and select the types of nonmarket practices (lobbying, campaign contributions, etc.) that best fit and align with their overall corporate objectives without neglecting the host market environmental culture. Research on market and nonmarket integrations have long been overdue given its extensive proposition to firm sustainable performance in a foreign country. Our research shed light on the importance of strategy integration to combat the ever-changing dynamism of the business environment and the negative sentiment surrounding globalization and how a firm can successfully compete in an uncertain, highly regulated, and standardized market context.

## Résumé

L'incertitude environnementale mondiale et la nécessité pour une organisation de maximiser ses profits et de satisfaire les intérêts de groupes/parties prenantes non marchands plus larges sur le marché hôte propulsent et renforcent la nécessité d'une intégration stratégique pour atteindre une performance d'internationalisation durable. L'intérêt de cet article est d'examiner l'impact relatif de la stratégie marchande, non marchande et de l'intégration stratégique sur la performance des moyennes et grandes organisations au Portugal. En outre, l'incertitude environnementale et les capacités de l'entreprise ont été utilisées comme modération pour évaluer l'implication de la performance de ces configurations de stratégie sur l'avantage du marché de l'entreprise dans le pays hôte. Étant donné la nécessité d'examiner la relation entre les variables latentes et mesurées dans cette étude, des modèles d'équations structurelles ont été utilisés pour tester les hypothèses énoncées, tandis que des analyses factorielles confirmatoires ont été utilisées pour évaluer l'adéquation de notre modèle. Nos résultats ont révélé que l'intégration de la stratégie offre une performance concurrentielle durable par rapport à Marché ou Stratégie hors marché lorsqu'ils sont utilisés séparément, en particulier dans des contextes commerciaux hautement réglementés et standardisés tels que le Portugal. De plus, nos résultats montrent que l'organisation doit concevoir des stratégies axées sur le marché et sélectionner les types de pratiques non marchandes (lobbying, contributions aux campagnes, etc.) qui correspondent le mieux et s'alignent sur leurs objectifs généraux d'entreprise sans négliger la culture environnementale du marché hôte. La recherche sur l'intégration marchande et non marchande est attendue depuis longtemps compte tenu de sa proposition étendue de performance durable de l'entreprise dans un pays étranger. Notre recherche a mis en lumière l'importance de l'intégration de la stratégie pour lutter contre le dynamisme en constante évolution de l'environnement des affaires et le sentiment négatif entourant la mondialisation et comment une entreprise peut être concurrentielle avec succès dans un contexte de marché incertain, hautement réglementé et standardisé.

## Summary highlights

### Contributions of the paper

We contribute to the field of nonmarket strategy by showing the performance implication of strategy integration (MS-NMS) on firm internationalization success and also depict the role that environmental uncertainty and firm capabilities play in this strategy conundrum/configurations. This research is the first to depict these interactions and interrelationships.

### Research questions/purpose

The purpose of the study is to examine the concurrent interaction between market and nonmarket strategies through a strategic integration framework for sustainable internationalization performance.

### Methodology

The study adopts structural equation model to test the stated hypotheses and confirmatory factor analysis to examine the fitness of the study model.

### Data base/information

This is a cross-sectional research design that explores the relationship between the latent and measured variables in this study (market, nonmarket, strategy integration, environmental uncertainty, and firm capabilities) through a stratified sampling technique. The study collected data from medium and large multinationals corporations in Portugal.

### Results/findings

Findings revealed that strategy integration provides a more sustainable competitive performance than either market or nonmarket strategy when used separately, especially in highly regulated and standardized business contexts such as Portugal. Similarly, our findings show that organization needs to design market-oriented strategies and select the types of nonmarket practices (lobbying, campaign contributions, etc.) that best fit and align with their overall corporate objectives without neglecting the host market environmental culture.

### Limitations (if there is any)

We recognize two limitations in this study. First, we collected data from different (medium and large) MNEs without paying significant attention to peculiar industry characteristics which may have an impact on MS, NMS, and strategy integration approaches adopted by these firms. Second, data from this study were subjected to firms with operational presence in Portugal. Hence, operations in other European countries, the USA, and the UK were not included.

### Managerial/theoretical implications and recommendations for future research

Following the logic of the behavioral theory of the firm and stakeholders theory used in this study, it shows that organizations will adopt CPA practices such as (lobbying, campaign contributions, and donations among others) to complement market strategy to enhance internationalization performance (improve market and competition and customer satisfaction) especially when international market performance and profit maximization is below aspiration level.

### Practical implications and recommendations

Managers need to understand that the same level of resources and planning required for engaging market-oriented strategies is quite similar to that required for engaging nonmarket strategies. This is why most multinational firms view MS and NMS as complementary, hence the emphasis on strategy integration. Also, managers need to work on developing and strengthening their capability configurations to create and capture sustainable market-oriented strategies for organizations. This is because of the multilayered interactions’ effect of environmental uncertainty in a developed and developing market context which may harm a firm sustainable market advantage when firms do not regard strategy integration.

### Suggestions for future research avenues

We encourage future researchers to engage in longitudinal research design given its detailed orientation to uncover the long-term impact of strategy integration on firm internationalization performance. Similarly, the relationship between firm size, firm characteristics, strategy integration (MS-NMS), and firm performance requires further research. This is probably because of resource discrepancies which may affect the response rate of medium and large organizations to NMS practices.

## Introduction

The concept of market and nonmarket integration was first mentioned in literature by Baron (1995) who opines that successful internationalization hangs on strategic integration of market and nonmarket strategies (NMS) into a firm business framework. Prior to now, market strategies (MS) have been a prominent force in crafting and leveraging the international market, given its extensive focus on competitors and the deployment of firm capabilities to enhance firm performance (Salavou 2015). Research on market strategies has been very novel given its extensive focus on managing customers/suppliers’ expectations, competitors’ actions, and inaction using either differentiation, cost-leadership, and other market-oriented competitive strategies to influence the desired market performance (Madanoglu et al. 2014). However, the uncertainty surrounding the international business environment, especially in the wake of the recent COVID-19 pandemic, has weakened the effectiveness and the sole use of market strategy in an international context giving prominence to the emergence and the need for strategy integration (MS-NMS) into a firm business framework (Gonzalez-Rodriguez et al., 2018; John and Lawton 2017; Charoensukmongkol 2022). Scholars have argued that the dynamic competitive business environment makes it impossible for a firm to sustain a market-oriented performance strategy on a long-term basis (Oliver and Holzinger 2008; Barney 2014; Kim, 2022), hence the need for strategy integration (MS-NMS) emphasized in this study. Nonmarket strategies (NMS) refer to the tactics adopted by organizations especially multination corporations (MNEs) to influence host country strategic policies through lobbying, campaign contributions, and setting up a political action committee (PAC) among others to the advantage of an organization.

Uncertainty in the business environment may force changes in firm services, production arrangements, customer-demand and technology adoption, and organizations’ needs to craft sustainable strategic responses (MS-NMS) to survive and achieve competitive advantage in the host market (Arora et al. 2016; Parnell 2018; Vaitoonkiat and Charoensukmongkol 2020a, b). The work done by Zhang et al. (2020) shows that there is a relationship between market uncertainty, firm innovation, and high firm performance. This shows that environmental uncertainty may act as a driver of strategic change which may or may not affect the competitive positions of firms depending on the strategy configurations in the host market. Similarly, anti-globalization sentiments and the global environmental uncertainty give credence to strategy integration as firms now deploy resources to influence policies in the favor of organizations. Empirical findings have established a significant positive relationship between MS and firm performance while NMS and firm performance linkage are currently growing (see Köseoglu et al. 2013; Parnell 2018; Parnell and Brady 2019; van Kranenburg and Voinea 2017; Zhang et al. 2020); however, literature on MS-NMS (strategy integration) configuration to enhance firm internationalization performance remains scanty. Hence, this research provides insight and expands knowledge on the importance of strategy integration (MS-NMS), especially in uncertain/volatile international market contexts.

Organizations are incorporating NMS emphasis (understand stakeholders and leverage policies) into their strategic business framework to complement market strategy for successful internationalization performance (Yin et al. 2016; Parnell 2018). NMS research and particularly corporate political activities (CPAs) have gained significant momentum in literature as organizations are integrating CPA practices into their strategic business framework, and this has led to the linkage of NMS with firm performance (Dorobantu et al. 2017; Parnell and Brady 2019; van Kranenburg and Voinea 2017). Similarly, organization emphasis on NMS is factored toward understanding the political, legal, social, environmental, regulatory-institutions, interest-groups, and media-agency embedded in international context each of which may assert policies and influence the success and sustained competitive position of firms especially in an uncertain/volatile international market contexts (Köseoglu et al. 2020).

Furthermore, the resource-based view (RBV) perspective emphasizes the importance of firm resources and dynamic capabilities as a useful tool for positioning and competing in a turbulent and uncertain business environment, but how these capabilities can be used to enhance performance under a strategy integration framework remains unsettled (Wei et al. 2015a, b, c). Similarly, Parnell (2018) examined strategic capabilities with specific reference to marketing and management capabilities and their relative effects on NMS practices of the firm but failed to capture the integrative effect of MS-NMS linkage and how it can drive high-performance advantage in an uncertain/volatile market contexts.

Thus, based on a survey conducted among the top management staff of multinational corporations (MNEs) in Portugal, our findings show that a firm that uses strategy integration performs better and has a higher chance of achieving sustainable internationalization performance than a firm that competes separately with either strategy. Our findings contribute to NMS literature in the following ways: first, the study responds to calls and suggestions made by Baron (1995), Parnell (2018),and Zhang et al. (2020) on the need for strategy integration, especially in a developed market context. Secondly, the study provides insight and understanding of the moderating effect of environmental uncertainty and firm capabilities on the need for strategy integration and helps to explore and understand the changing conditions of this strategy conundrum.

## Literature review

### Nonmarket strategies, market strategies, and strategy integration

Research on nonmarket strategies cuts across several disciplines ranging from political sciences, political economics, and or strategic management. In general, studies coming from political economics assume that organizations engage in politics to influence political institutions’ policies in the favor of the organization (Wrona and Sinzig 2018), while studies coming from political science argue that the sole determinant of competition among interest groups is due to public policy; thus, firms engaging in politics may weaken public interest and undermine democratic practices (Wrona and Sinzig 2018). However, scholars from the fields of political sciences and political economics view NMS practices from the angle of industry-level perspective thereby neglecting firm-specific features and structures that are important to CPA practices (Lawton et al. 2013; Dorobantu et al. 2017), whereas scholars coming from the strategic management field have consistently paid attention to firm-specific advantages as a driver of CPA behavior (Oliver and Holzinger 2008; Köseoglu et al. 2020). Conversely, scholars on CPA behavior have come to a common ground concerning firm-level characteristics essential for driving NMS activity in organizations. These characteristics include the size of the firm, slack resources, and the pertinent issues that firm faces. Empirical findings have shown that large organization tends to have the required resources to engage more in political NMS practices (Bonardi et al. 2006; Meyer and Peng,^ 2016^; Charoensukmongkol 2016). Research has also established that firms engage in CPA practices or political corporate social responsibility (PCSR) depending on the issues that are pertinent to the firm (Liedong et al. 2017).

However, studies on market strategies (MS) have particularly focused on customers, competitors, demand, and supply with emphasis on market or competitive strategies such as cost leadership, differentiation, and other competitive tactics, all of which are factors that determine the market and competitive positions of organizations in the international market contexts (van Raaij and Stoelhorst 2008; Köseoglu et al. 2020). For instance, in a saturated and or highly concentrated industry where policy changes are eminent, to ensure standardization, a firm may use cost leadership or differentiation strategies to create a market and competitive niche and enhance customer satisfaction. NMS involves political actions undertaken by firms to influence host government political institutions’ policies, generate social advantage (PCSR), and gain stakeholder legitimacy (Mellahi et al. 2016; Liedong et al. 2017). Market actions/strategies are essentially directed towards influencing buyers (customers), sellers, and market and competitor actions and inaction. For instance, developing new product, engaging in a price war, promotion, advertisement, strategic alliance, expansion, and diversification among others are all essential market strategies that can be deployed to influence customers’ decisions towards the organization’s product and services (Ferrier et al. 1999; Dewnarain et al. 2019), whereas NMS practices commonly used are lobbying and political action committee (PAC) (Lawton et al. 2013; Delmas and Montes-Sancho 2010); others include campaign contributions, constituency building, and donations/bribery among others (Doh et al. 2012; John and Lawton 2017).

Additionally, NMS research shows that firms engage in CPA behavior or PCSR for value maintenance or value creation; either way, protecting the value system of a firm is germane to firm engagement in NMS practices in the international contexts (Oliver and Holzinger 2008; Wei et al. 2015a, b, c). Value maintenance has been described in the literature as “domain defense” or “defensive-political-strategies,” while value creation is referred to as “domain maintenance” or “proactive political strategies (Oliver and Holzinger 2008; Wei et al. 2015a, b, c). Therefore, an organization will do everything possible to protect and maintain its value proposition for the international market, hence the need to integrate NMS emphasis into a firm strategic business framework for the international market. Interestingly, the assumption of two management theories strongly supports the importance of strategy integration for the international market contexts. First, behavioral theory of the firm assumes that an organization will always seek profit maximization through effective utilization of its resources and cognitive capabilities when taking business risks especially when performance expectations are yet to be achieved in the organization (Ji-Yub et al. 2011; Liu et al. 2015). Thus, low-profit maximization and or performance below firm aspiration level for the international market may force organizations to integrate NMS practices into their business framework, thereby enhancing internationalization performance. Second, stakeholder theory emphasizes the need for organizations to focus on a broad range of groups and institutional interests other than suppliers, customers, and competitors that may influence the operations of firms in the international market (Hillman and Keim 2001; Parnell 2018; Vaitoonkiat and Charoensukmongkol, 2020). This shows that the anti-globalization sentiments and the global environmental uncertainty placed great importance on the need for an organization to think beyond satisfying the need of international market consumers/suppliers or shareholders’ interests alone but also meets the interest of important stakeholders in the host market.

## Hypotheses

### Market strategy and firm performance

As stated above, the focus of MS is mainly on managing the action and inaction of competitors, suppliers, and customers in order to improve and maintain a firm competitive position in the market (Wei et al. 2015a, b, c). For instance, when a firm engages in a price war, promotion and advertisement, portfolio diversification, strategic alliance, and new service development among others, all of these reflect the actions taken by firms to manage suppliers, customers, and competitors’ relationships within an industry (Dewnarain et al. 2019). According to Porter and Kramer (2006), firms can design competitive strategies of low cost and or differentiation strategy to compete favorably in the market. Empirical research on competitive strategies (low cost and differentiation) and firm performance has been growing in literature (Parnell 2018; Zhang et al. 2020). The major goal of firms is to overtake their rivals by consistently using low cost or differentiation strategies to create a sustainable competitive niche, thereby meeting the needs and wants of customers.

Research on competitive strategies and environmental uncertainty has been very supportive, mainly because empirical findings have shown a positive link between firm innovation, high firm performance, and environmental uncertainty (Zhang et al. 2020; Parnell 2018; Vaitoonkiat and Charoensukmongkol, 2020). For instance, uncertainty pushes firm to seek new and/or improved ways to produce products and services and meets customer needs and wants in a more efficient way (Porter and Kramer, 2006; Dewnarain et al. 2019). This shows that the strength of firm performance and innovative business services are sometimes influenced by how a firm can explore market uncertainty through firm capabilities to achieve high firm performance. Environmental uncertainties have multilayered interaction effects on an organization’s performance which is mostly based on firm dynamic capability development and strategy configurations for the host market (Dahan et al. 2013). This implies that some organizations may interpret environmental uncertainty as an opportunity to innovate and develop new products and services to meet the changing environmental conditions, while others may see it as a disadvantage (Hadani and Schuler 2013). However, scholars argue that firm dynamic capability development plays an essential role in this interpretation and interaction. Thus, considering the positive link between MS performance linkages in literature, we propose two dimensions of performance (market and competition, and customer satisfaction) for this study, where market and competition refer to all the competitive strategies/actions of an organization to create and maintain a competitive position and enhance customer satisfaction in the market while a decrease in customer complaints leading to customer satisfaction is another key performance emphasis. Hence, we anticipate a positive association in this study between the two dimensions of organizational performance.

- H1a: Emphasis on market strategy will improve market and competition
- H1b: Emphasis on market strategy will improve customer satisfaction
- Ho1c: Environmental uncertainty positively moderates the association between market strategy and market and competition
- Ho1d: Environmental uncertainty positively moderates the association between market strategy and customer satisfaction

### Nonmarket strategy and environmental uncertainty

As earlier stated, the issue of environmental uncertainty is multifaceted. It relies heavily on the manager’s competence and the strength of the organization’s internal capabilities. For instance, most organizations rely on environmental uncertainty to create a competitive niche thereby enhancing the financial and non-financial performance of the organizations (Qian et al. 2016; Wang et al. 2015). Managers can leverage environmental uncertain markets to produce new products and services and meets customer needs and wants, thereby improving customer satisfaction, enhancing technology adoption, entering new markets, and improving firm value chains (Qian et al. 2016; Köseoglu et al. 2020). Hence, the greater the environmental uncertainty, the greater the emphasis on NMS practices and strategy integration by firms (Chen et al. 2016; Wilden and Gudergan 2015). As an example, the best response to high environmental uncertainty for some firms is to create product or process innovation, while for others, the best response is to increase their NMS practices by increasing their political affiliation (Charoensukmongkol 2016). In the same way, a market characterized by low environmental uncertainty will likely experience a low emphasis on NMS and the need for strategy integration (Glynn and Abzug 2002; Marquis et al. 2007; Parnell 2018). Environmental uncertainty makes market strategies less relevant and gives prominence to CPA practices such as lobbying legislators to restrict entry barriers, using CSR strategy to enhance public interest and firm legitimacy and or seeking a government loan.

If successful, the firm may even leverage political affiliation to reduce environmental uncertainty on technology adoption and competition by erecting barriers to competitive action that may affect the firm competitive position. Scholars argue that the relationship between environmental uncertainty and firm performance is subjective because it depends solely on the managers’ view, internal capabilities readiness, and the interpretation given of market uncertainty by the firm (Barney 2014; Parnell 2018). However, empirical support shows that environmental uncertainty has a significant positive impact on firm innovation and performance (Parnell 2018; Zhang et al. 2020). A business environment characterized by high uncertainty places great emphasis on firm capability development and may render existing knowledge and resources obsolete, thereby requiring consistent new product development which may shorten the existing product life cycle. Therefore, a firm must constantly assess these threats and restructure its capabilities to be able to manage the business competition. On this premise, we assert that environmental uncertainty can be said to have a great influence on firm’s innovative performance, and thus, we expect that environmental uncertainty will have a positive relationship with the dimensions of firm performance and the adoption of NMS practices in this study.

- H2a: Emphasis on nonmarket strategy will improve market and competition
- H2b: Emphasis on nonmarket strategy will improve customer satisfaction
- Ho2c: Environmental uncertainty positively moderates the association between nonmarket strategy and market and competition
- Ho2d: Environmental uncertainty positively moderates the association between nonmarket strategy and customer satisfaction

### Strategy integration (MS-NMS) and firm capabilities

Integration refers to the independent and concurrent use of both MS and NMS strategies for a sustainable competitive position. Literature on strategy integration in international business is still developing and very fragmented in relation to firm performance. Scholars argue that firms will prefer to use proactive nonmarket strategies (lobbying, campaign contributions, political action committee among others) to mitigate the sociopolitical pressures than a reactive nonmarket strategy (operational compliance, capability development, etc.), especially in a volatile/uncertain international market context (Meznar and Nigh, 1995; Du, Bai and Chen, 2019). This implies that firm will easily adopt strategy integration to mitigate and curb market and nonmarket social pressures from the host market. Additionally, the integrative interaction between market and nonmarket practices is expected to yield a negative relationship by international business scholars owing to the conflicting expectations of the market (consumers) and competitors and the nonmarket stakeholders. As an example, Barnett (2007) states that the CPA engagement through lobbying practices and campaign contributions among others is mainly aimed toward enhancing stakeholders’ relationships for favorable policy implementation but may necessarily not satisfy consumers’ expectations of organization products and services in the host market.

Conversely, empirical findings have been very supportive of strategy integration. For instance, Wei et al. 2015a, b, c) found that rival response and speed of market retaliation significantly reduced when a firm adopts MS-NMS integration into its business framework than when either strategy was used separately. Shaffer, Quasney, and Grimm (2000) found positive empirical support showing integrated MS-NMS action and firm performance among the data collected from the airline’s industry covering the international route. Over the years, scholars have stressed the significant outcomes of adopting an integrated strategy (Bonardi et al. 2006; Ferrier et al. 1999), although Baron (1995) and Mahon and Waddock (1992) argue that the issue that a firm needs NMS practices to resolve is mostly caused by MS activities. Nevertheless, integrating MS and NMS strategies into a firm business framework increases the chances of firm survival and sustainable competitive advantage in the international market. For instance, Clougherty (2003) stated that firms interested in internationalization through mergers and acquisitions are sometimes faced with antitrust laws among others but have to integrate nonmarket strategies to get some antitrust exemptions in the international market.

**Fig. 1:**
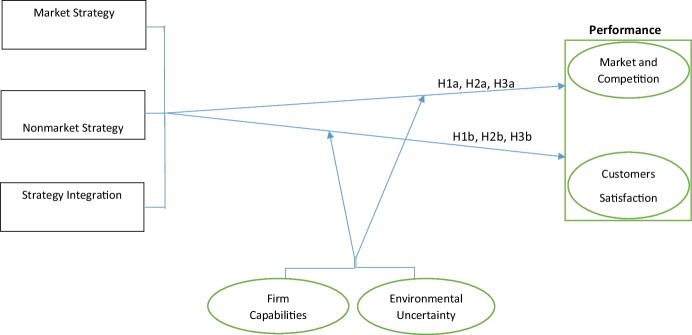
Research conceptual model

Furthermore, behavioral theory of the firm and stakeholder theory strongly supports the notion of strategy integration for the international market. For instance, behavioral theory of the firm assumes that profit maximization is the ultimate focus of an organization, and the organization will do everything possible to maintain and enhance its value proposition for the international market including engaging and lobbying host government political structures for effective internationalization performance (Ji-Yub et al. 2011; Liu et al. 2015). This shows that profit maximization is the ultimate goal of all organization international market investment, but when the desired goal is below the aspiration level, organizations will seek strategic means (e.g., lobbying) to influence host market performance. Similarly, stakeholder theory suggests that organizations should aim to satisfy a broad range of interest groups in the international market including political and institutional interests, suppliers, customers, and competitors’ interests as each of these interest groups’ policies may affect the competitive position of an organization in the host market (Hillman and Keim 2001; Parnell 2018). This implies that both the market and nonmarket interests groups must be simultaneously satisfied for sustainable market performance to be achieved. On this notion, we conclude that firms favoring strategy integration would perform better than those using either MS and or NMS strategies separately (Fig. 1).

- H3a: Emphasis on integrated MS-NMS strategy will improve markets and competition
- H3b: Emphasis on integrated MS-NMS strategy will improve customer satisfaction
- Ho3c: Environmental uncertainty positively moderates the association between integrated MS-NMS strategy and market and competition
- Ho3d: Environmental uncertainty positively moderates the association between integrated MS-NMS strategy and customer satisfaction
- Ho3e: Firm capabilities positively moderate the association between integrated MS-NMS strategy and market and competition
- Ho3f: Firm capabilities positively moderate the association between integrated MS-NMS strategy and customer satisfaction

## Methodology

The research instruments were designed and administered online using survey monkey to the management staff of selected multinational corporations in Portugal. The survey was sent to over 1900 management staff who are decision-makers in these organizations, and only 302 responses were received and used for our analysis. Management staff from these multinational corporations’ data were assessed from (BoldData.nl) services. BoldData.nl is an organization that provides data services (managers, CEO, among others) across several industries to facilitate SME businesses integration, new market development, and these data can be used for academic purposes. Also, Portugal’s economy was chosen as our sampling scope for the following reasons: Portugal’s economy after the 2013 economic crisis made several attempts to encourage entrepreneurship and enhance foreign direct investment (FDI) by implementing numerous policies aimed at cost reduction (cheap labor cost) for setting up businesses, seed financing for new firms, and creating an entrepreneurial ecosystem among others (Braguinsky et al. 2013). Nevertheless, the increasing birth of most of these new firms is now proportional to the mortality rate experienced in the country. These happened for two reasons: first, most of these start-ups are unable to experience actual growth given the influx of product market competition. Between 2010 and 2014, 41% of these new firms were required to pay in interest more than the company’s annual generated income without considering tax evasion and other operational financial obligations as peculiar to each firm (Dias et al. 2015).

The resource misallocation and lack of standardization gave rise to a high exit barrier and mortality rate. Second, discrimination exists between medium and large firms concerning labor law which serves as a disincentive to growth for these new firms. Only a few firms in Portugal can boost more than 50 employees, and this is due to inefficient labor law and resource misallocation (Lentz and Mortensen 2008; OECD 2015). To resolve some of these issues, efforts were made to enhance firm standardization and regulations which however led to excessive business regulations, strenuous environmental licensing, changing tax conditions, and new levies among others (Portugal Statistics 2015). These changes in labor law and firm standardization made the economy home to many MNEs as the country is now seen as a low labor cost country that possesses a high-skilled workforce. Furthermore, the country’s foreign direct investment started gaining significant momentum in 2014, and as of May 2022, the country’s FDI had a $1.2 billion increase which is compared to a $370.2 million increase in April of 2022. The influx of these multinational corporations in Portugal and the suggestion made by Parnell (2018) justifies the need to conduct the study in Portugal and to understand how MNEs are battling with the market and nonmarket policies.

Following the work of So et al. (2016), the online survey was appropriate to gather data for this study considering the restriction and lockdown that started in 2020 in Portugal. The justification for selecting management staff was due to the technicalities required in understanding and providing relevant answers to each of our construct items in the survey, hence the reason for the stratified sampling technique adopted in the study. Also, we used a 5-point Likert scale response type (i.e., 1 = greatly improved performance, 5 = deteriorated performance significantly) for each construct item in our survey. A structural equation model (SEM) was used to test our hypotheses, and confirmatory factor analysis was used to determine the fitness of the model. SEM was appropriate for testing our hypotheses because it is capable of showing the relationship between measured and latent variables (market, nonmarket, strategy integration, environmental uncertainty and firm capabilities) in the study (Hair et al.  2012). The survey instrument was made available in both English and Portuguese for ease of understanding to the managers. The survey distribution took approximately 2 months and 1 week to gather 302 responses from top-level management staff. Medium- and large-scale enterprises were included in the data collection which shows validation and credibility from a diverse pool of knowledgeable managers about the market and nonmarket strategic issues faced in their organization (Balogun and Johnson 2004). The survey instruments were validated by three professors of international business, and the pilot study results used to assess the reliability and validity of the instrument show 0.88 which exceeds the threshold of 0.70 recommended by (Nunnally, 1978).

## Measures

### Market strategy and firm performance

The contents of our survey were adapted from validated scales from the literature. For instance, items on market strategy were guided and assessed from (Nayyar 1993; Parnell 2018; Parnell and Brady 2019) work. A 7-item scale of market strategy was constructed, but only four items fit our model (α = 0.750; AVE = 0.564), while organization performance measures (market and competition, and customer satisfaction α = 0.807; AVE = 0.509) were inspired by multiple sources (Madanoglu et al. 2014; Norreklit 2000; Kaplan and Norton 2001, 2004; Harris and Mongiello 2001; Phillips 1999, Phillips and Moutinho 1999). Market and competition here refer to all the competitive strategies/actions of an organization to create and maintain a competitive market advantage through a focus on customer satisfaction while a decrease in customer complaints and a corresponding increase in customer satisfaction enhance customer retention in the international market which leads to sustainable internationalization performance.

### Nonmarket strategy

Measures of nonmarket strategy used to capture the political, social, and legal contexts of host country were adapted from (Deng et al. 2010; Hillman and Hitt 1999; Parnell and Brady 2019) taxonomy and extrapolated into four items through our measurement model (α = 0.851; AVE = 0.662). The measures of nonmarket strategy examined in this study include lobbying, campaign contribution, donation, government membership on the company board, political corporate social responsibility (PCSR), and political action committee (PAC) among others. These measures highlight how organizations are engaging with the host government’s political structure to influence favorable business policies.

### Strategy integration

Scale on strategy integration (MS-NMS) were adapted from (Baron 1995; Wei et al. 2015a, b, c; Wei et al. 2015a, b, c) model who opines that integration occur when firm uses both market and nonmarket strategies to resolve a particular market or nonmarket issues (α = 0.785; AVE = 0.592). Hence, we affirm integration when respondents choose a market and nonmarket actions to resolve a particular issue during our survey (see Tables 1, 2 and 3).

**Table 1:** Sample demographics

Variable	*n*	%
Gender		
Male	**194**	**64.2**
Female	**108**	**35.7**
Management level		
Top level	**149**	**49.3**
Middle level	**153**	**50.6**
Host country presence		
Headquarter	**96**	**31.7**
Subsidiaries	**206**	**68.2**
Industry		
Manufacturing	**85**	**28.1**
Services	**98**	**32.4**
Hospitality	**66**	**21.8**
Agriculture	**47**	**15.5**
Others	**6**	**1.9**
Functional background		
HR/general manager	**79**	**26.1**
VP public affairs/CSR	**102**	**33.7**
Legal	**81**	**26.8**
Member BoD/BoT	**40**	**13.2**
Firm size		
Large (301 + employees)	**181**	**59.9**
Medium (21–300 employees)	**121**	**40.0**

**Table 2:** Survey items—market strategy, nonmarket strategy—performance

*Item*	*Loading*	*Wording*
*Emphasis on market strategy (α* = *.750, composite reliability* = *.712, AVE* = *.564)*		
EMS1	0.841	Managing raw material cost
EMS3	0.827	Operating efficiency
EMS4	0.832	Product price reduction
EMS5	0.987	Strict product quality compliance
*Emphasis on nonmarket strategy (α* = *.851, composite reliability* = *.809, AVE* = *.662)*		
ENMS1	0.922	Lobbying government policies favorable to the organization
ENMS2	0.932	Contributing to societal values through various CSR strategies
ENMS3	0.910	Collaborating with government agencies to ensure standardization and restrict entry barriers
ENMS4	0.859	Setting up a political action committee to advance the company interests
Performance—markets and competition (*α* = *.807, composite reliability* = *.918, AVE* = *.509*)		
PMC1	0.856	Competitive niche
PMC2	0.758	New product and service development
PMC4	0.844	Overall firm performance
PMC5	0.856	Knowledge of competitors strength and weakness
*Performance—customers (α* = *.781, composite reliability* = *.851, AVE* = *.634*)		
PC1	0.989	Reduced customer complaints
PC3	0.926	Customer satisfaction
PC5	0.897	Customer retention

*Note: AVE a*verage variance extracted*, α* = *Cronbach alpha*

**Table 3:** Survey items—environmental uncertainty, firm capabilities, and MS-NMS integration

*Item*	*Loading*	*Wording*
*Integration MS-NMS (α* = *.785, composite reliability* = *.961, AVE* = *.592)*		
Integration1	0.756	EMS1–ENMS1
Integration2	0.968	EMS4–ENMS3
Integration3	0.744	EMS3–ENMS2
*Firm capabilities (α* = *.720, composite reliability* = *.835, AVE* = *.602)*		
FC1	0.828	Knowledge of customers changing taste
FC3	0.819	Capabilities of creating durable relationship with our suppliers
FC4	0.811	Ability to predict technological changes in the industry
FC5	0.812	Cost control capabilities
*Environmental uncertainty (α* = *.864, composite reliability* = *.815, AVE* = *.615*)		
EU2	0.724	Nonstop changing product preference of customers
EU3	0.912	Creating new product ideas through technological breakthroughs
EU5	0.783	We engage in promotional wars

*Note: AVE* average variance extracted*, α* = *Cronbach alpha*

### Moderator (environmental uncertainty and firm capabilities)

The view of environmental uncertainty is dynamic and multifaceted and mostly reliant on firm dynamic capabilities. Our 5-item scales were adapted from Jaworski and Kohli (1993) and Desarbo et al. (2005) and supported by α = 0.864; AVE = 0.615, while the 6-item scale on firm capabilities (α = 0.720; AVE = 0.602) were assessed from Desarbo et al. (2005). These moderators highlight how an organization can strategically develop and position its capabilities among host market stakeholders for effective internationalization performance.

## Results measurement model

To test our hypotheses, we first checked some important assumptions in our model to determine the model fitness, such as multicollinearity, sample size, missing values, normality, reliability and validity, and common method bias (Kline 2005). Our sample size was 302; therefore, we do not violate the recommended value of 200 before carrying out a structural equation model (Iacobucci 2010) to test our hypotheses. On normality, we checked for skewness and kurtosis from all our questionnaire items, and the values were within the suggested threshold of − 1 to + 1 (Bagozzi and Yi 2012). Multicollinearity was assessed using correlation analysis. A value above 0.5 is deemed good, and all our variables have value above 0.5 (Field 2005). All our scales were checked for reliability and validity, and the results show that Cronbach alphas exceeded 0.7, composite reliability exceeded 0.8, and average variance extracted exceeded 0.5 for all our construct as recommended by Bagozzi and Yi (2012) (see Table 2). Finally, we check for common method bias first, by assuring respondent data anonymity and differentiating between independent and dependent variables for easy understanding. Second, the data was collected from multiple sources knowing fully well that collecting data from one single source could result in common method bias (Podsakoff et al. 2012). Third, following Harman’s single‐factor test, our results show chi-square = 31.347, IFI = 0.65, CFI = 0.62, TLI = 0.53, NFI = 0.67, and RMSEA = 0.28; hence, a single factor was not responsible for majority of the variance in our data. Therefore, common method bias was not a problem in this study.

## Findings

Before testing our model, we assessed the general fitness of the model using confirmatory factor analysis. A good fit is measured using goodness of fit index (GFI), comparative fit index (CFI), normed fit index (NFI), Tucker-Lewis index (TLI), and root mean square error of approximation (RMSEA) (Guimaraes et al. 2016; Bagozzi and Yi 2012). Our results reveal that the fit statistic using the recommended threshold was acceptable as (i.e., χ2 = 2.412; GFI = 0.920; TLI = 0.943; CFI = 0.912; NFI = 0.914; and RMSEA = 0.073). All the hypotheses were tested using a structural equation model with a bootstrapping indicator set at 2000 for the moderators (environmental uncertainty and firm capabilities) to determine the extent of influence and indirect effect on each of the constructs. R2 coefficient for firm capabilities, environmental uncertainty, market and competition, and customers are 0.626, 0.270, 0.916, and 0.857, respectively. Strategy integration was achieved by selecting the number of times respondents agreed that the firm took a market and nonmarket simultaneous action as a competitive strategy in their organizations to achieve particular market advantage. Thus, following Wei et al. (2015) integrated model formulation, the following market and nonmarket competitive actions (EMS1-ENMS1, EMS4-ENMS3, and EMS3-ENMS2) were mostly used concurrently by sampled respondents in their firms (see Table 3).

Findings for the direct hypotheses show that *H1a* and *H1b* were supported (see Table 4); that is, emphasis on market strategy contributes significantly to market and competition and customer satisfaction. Similarly, *H2a* was supported whereas *H2b* was not supported, showing that emphasis on NMS contributes significantly to market and competition but not directly to customer satisfaction and also shows a negative relationship. Interestingly, *H3a* and *H3b* were supported, showing that integrated MS-NMS actions contribute significantly to markets and competition, and customer satisfaction which leads to sustainable internationalization performance.

**Table 4:** Result of hypotheses testing—direct effect

*Hypothesis*	*Path*	*β*	*t value*	*p*	*Remark*
H1a	MS → MC	0.216	3.832	***	Supported
H1b	MS → CS	0.376	4.628	***	Supported
H2a	NMS → MC	0.225	3.527	***	Supported
H2b	NMS → CS	− 0.185	− 1.268	0.07	Not supported
H3a	MS-NMS → MC	1.114	5.225	***	Supported
H3b	MS-NMS → CS	1.122	8.601	***	Supported

Note(s): significant at *p* < 0.05

On the moderation effect, *H01c* was supported but *H01d* was not supported (see Table 5), showing that environmental uncertainty positively moderates the relationship between market strategy, market, and competition but not with customer satisfaction and likewise shows a negative relationship. Also, *H02c* was supported but *H02d* was not supported, showing that environmental uncertainty positively moderates the relationship between NMS and market and competition but not customer satisfaction and also shows a negative relationship. Interestingly, *H03c, H03d, H03e*, and *H03f* were supported, showing that both environmental uncertainty and firm capabilities significantly moderate the relationship between integrated MS-NMS, market and competition, and customer satisfaction. However, the moderated relationship between environmental uncertainty and MS-NMS and customer satisfaction shows a negative relationship.

**Table 5:** Result of hypotheses testing—moderation effect

*Hypothesis*	*Path*	*Effect*	*Lower bound*	*Upper bound*	*p*	*Remark*
Ho1c	MS → EU → MC	.145	.113	.045	.002	Supported
Ho1d	MS → EU → CS	− .002	− .005	− .007	.106	Not supported
Ho2c	NMS → EU → MC	.189	.069	.050	.001	Supported
Ho2d	NMS → EU → CS	− .004	− .002	− .006	.136	Not supported
Ho3c	MS-NMS → EU → MC	.514	.024	.035	.024	Supported
Ho3d	MS-NMS → EU → CS	− .409	− .018	− .025	.000	Supported
Ho3e	MS-NMS → FC → MC	.313	.011	.064	.004	Supported
Ho3f	MS-NMS → FC → CS	.614	.014	.032	.001	Supported

Note(s): significant at *p* < 0.05

## Discussion

The call for strategy integration especially for multinational organizations rekindles long-term debates on how a firm can achieve and maintain sustainable competitive advantage regardless of a firm and industry-level competitive factors (Baron 1995; Bonardi 2004; Schuler et al. 2002; Shaffer and Hillman 2000; Parnell 2018). This call reveals the weakness of firms competing separately with either market and or nonmarket strategies, especially in today’s dynamic business environment. Responding to the call, our findings show that strategy integration helps a firm to achieve and maintain sustainable firm performance without neglecting the individual relationship that market and nonmarket strategies have on firm performance (Henisz and Zelner 2012; Cavazos and Rutherford 2012; Vázquez-Maguirre and Hartmann 2013; Mellahi et al. 2016). We found a similar notion in Wei et al. (2015) who found that rival response and speed of competitive actions or retaliation in the market were very low when firms adopt integrated actions and high when firms adopt either MS or NMS separately in the international market.

Our results show that market strategy has a significant impact on firm performance (market and competition, and customer satisfaction), and this aligned with the findings of scholars on similar constructs (Köseoglu et al. 2013; Parnell 2018; Zhang et al. 2020). These findings show that regardless of the complementary nature of NMS practices in today’s dynamic business environment, the importance of market-oriented strategies to firm performance cannot be downplayed, although the moderating effects of environmental uncertainty on the relationship between market strategies and customer satisfaction were not supported and even show a negative relationship. This, however, is very intriguing given the findings of Köseoglu et al. 2013, 2020) who found that organizations especially multinationals engage in market-oriented strategies such as differentiation and or cost leadership to compete favorably and differentiate their offerings in an uncertain market context. Nonetheless, the heterogeneous company selections for data gathering or the environmental context of Köseoglu et al. 2013, 2020) research might be responsible for the differences in findings.

Also, our findings show significant positive support between NMS practices and market and competition but show a negative insignificant relationship with customer satisfaction. These results are very intriguing but not surprising. As earlier stated, many of the issues that a firm needs NMS practices to resolve are created by market-oriented strategies. Also, the moderating effects of environmental uncertainty on the relationship between nonmarket strategies and market and competition were supported but customer satisfaction was not supported and even show a negative relationship. These findings also conflict with the position of Köseoglu et al. 2013, 2020) but take a cue from Kim (2022)’s work which states that NMS practices can influence directly market and competitive position of a firm but may have little or no direct influence on customer satisfaction. Similarly, Dewnarain et al. (2019) found that the satisfaction of customers is directly related to the customer experience of company products and services which can only be achieved through market-oriented strategies aimed at meeting customers’ needs and wants.

Interestingly, integrated actions of MS-NMS strategy show a significant positive relationship with firm performance (market and competition and customer satisfaction). We anticipated these results following the call for papers by Baron (1995), Parnell (2018), and Köseoglu et al. (2020) to statistically address strategy-integration-performance linkages in developed market contexts. Furthermore, we found that most MNEs in Portugal favor strategy integration due to the high level of business standardization and regulations as it enables organizations to influence policies that may affect their sustainable market advantages. Furthermore, the moderating effect of environmental uncertainty and firm capabilities on the integrated actions (MS-NMS) of organization and firm performance constructs examine in this study also affirms the importance of strategy integration for the international market. Environmental uncertainty is a firm worst challenge when market and nonmarket strategies are used separately and may act as an opportunity for a firm to differentiate its services and enhance its political and market capabilities when a firm adopts strategy integration. Recent findings by Parnell (2017) and Zhang et al. (2020) show that market uncertainty enhances a firm innovative business decision and market-oriented differentiation strategy, thereby improving firm performance. A firm needs to be able to develop political and market capabilities to enjoy a sustainable competitive advantage in a dynamic market context. Hence, environmental uncertainty regardless of whether high or low can be viewed from the strength of a firm competitive knowledge and capability development (Teece et al. 2016). Based on these findings, we, therefore, conclude that integrated actions are key to a firm sustainable competitive advantage and even support decades of a conceptual proposition on strategy integration in international business literature (Baron 1995; Schuler et al. 2002; Shaffer and Hillman 2000; Bonardi 2004).

## Managerial implications

Several important managerial action points can be lured out from our findings. First, managers need to work on developing and strengthening their capabilities configurations to create and capture sustainable market-oriented strategies for organizations (Parnell 2015; Köseoglu et al. 2013). This is because of the multilayered interactions effect of environmental uncertainty in a developed and developing market context which may harm a firm sustainable market advantage. The findings in this study reinforce the importance of capability development and provide insight into the relevance and ways it can serve as a differentiating factor for a firm in an uncertain market environment and under a strategy integration framework to improve firm performance (Wu et al. 2012; Theodosiou et al. 2012). Second, managers need to understand that the same level of resources and planning required for engaging market-oriented strategies is quite similar to that required for engaging nonmarket strategies. This is why most multinational firms view MS and NMS as complementary, hence the emphasis on strategy integration.

Although the difference exists in the level of financial resources commitment required for both MS and NMS engagement, this is why some managers favor market-oriented strategies over NMS practices (Hadani et al. 2015). Also, environmental contexts are another underpinning factor for managers in deciding between MS, NMS, and/or strategy integration; nevertheless, managers need to evaluate and assess the business needs in line with the prevalent host market realities and decide on the best NMS practices (e.g., lobbying, campaign contributions) to undertake that will drive sustainable market performance. Finally, MNEs in Portugal especially those in highly regulated industries (services, hospitality, and manufacturing) are favoring strategy integration to influence host government policies that affect market and nonmarket activities to create a sustainable market performance (Singer 2013; Frynas et al. 2017). Managers, therefore, need to understand the nature and culture of their industry businesses and the relative policy changes and strategically position firm activities to navigate the challenging market and nonmarket risks in the host market.

## Theoretical implications

Based on Baron (1995)’s proposition with support from Oliver and Holzinger (2008) and Parnell (2018), engaging with the host country’s political and regulatory structure provides a source of competitive advantage for the organization, because it allows an organization to withstand and benefit from the changing environmental conditions and continuously help to create and maintain their value proposition for the international market. Advancing the logic of the behavioral theory of the firm and stakeholders theory, it shows that organizations will adopt CPA practices such as (lobbying, campaign contributions, and donations among others) to complement market strategy to enhance internationalization performance (improve market and competition and customer satisfaction) especially when international market performance and profit maximization are below aspiration level (Du, Bai and Chen, 2019). Scholars have shown that organizations can manage effectively the relationship embedded with strategic nonmarket actors and as depicted by stakeholders’ theory that organizations must focus on satisfying a wider range of interest groups including political and host market regulatory structures in the international market. Following these assumptions, we argue that a firm should take a proactive position in pursuing strategy integration for the international market as it aimed to achieve a sustainable internationalization performance.

Furthermore, the strategic interaction between market and nonmarket practices not only depicts a distinct unseparated line of research especially due to the global environmental uncertainty (Mellahi et al. 2016) but additionally provides a holistic view of corporate NMS practices (Dorobantu et al. 2017), as it emphasizes on how an organization can create, maintain, and reconfigure numerous NMS practices to achieve sustainable competitive advantage in the international market. Although it is established knowledge that the factors affecting the changing political and regulatory conditions of the host market are extremely difficult to predict due to the cause and effect variations, they are not impossible to manage (Frynas et al. 2017). This implies that organization needs to be aware that they might not be able to generate all their returns on nonmarket investment. However, the integrated perspective emphasized in this study suggest that organization may need to choose some nonmarket approaches (e.g., lobbying, campaign contributions) that aligned with their corporate market objectives and strategically configure them to satisfy a broader interest group to maximize profit in the international market. Thus, our empirical findings and theoretical arguments demonstrate the importance of strategy integration as it depicts the benefits and difficulties embedded in engaging with host market political and regulatory structure and how organizations need to differentiate their market and nonmarket objectives for favorable international market advantage.

## Limitations and future research

We recognize two limitations in this study. First, we collected data from different (medium and large) MNEs without paying significant attention to peculiar industry characteristics which may have an impact on MS, NMS, and strategy integration approaches adopted by these firms. Second, data from this study were subjected to firms with operational presence in Portugal. Hence, operations in other European countries, the USA, and the UK were not included, although our study was a response to the suggestion for further study made by Baron (1995) and Parnell (2018) in developed European countries. Therefore, our findings are applicable to other European countries with highly regulated and standardized practices for entrepreneurship development. Nevertheless, future researchers can extend these findings to other developed and developing European countries to examine the impact that strategy integration has on firm internationalization performance in a regulated and less regulated market and industries.

Also, this study used a cross-sectional research design; thus, data were collected within a single period even though common method bias shows no significant impact (Kobrin 2015). We encourage future researchers to engage in longitudinal research given its detailed orientation to uncover the long-term impact of strategy integration on firm internationalization performance. Similarly, the relationship between firm size, firm characteristics, strategy integration (MS-NMS), and firm performance requires further research. This is probably because of resource discrepancies which may affect the response rate of medium and large organizations to NMS practices, even though the negative relationship between NMS, environmental uncertainties, and customer satisfaction in this study suggests that large organizations will prefer strategy integration as a way to curb environmental uncertainties given their latent resource advantage.

## Conclusion

Our findings show that MS, NMS, and strategy integration enhance firm performance (market and competition, and customer satisfaction) in different and unique ways (Köseoglu et al. 2020; Parnell 2018). We established before now that the relationship between market strategy and firm performance has long been established in the literature and our results show no discrepancies. However, we found that the relative direct impact of market-oriented strategies on customer satisfaction as examined in this study is not the same when compared to nonmarket strategies. This further shows the uniqueness of the two approaches/strategies to firm performance and reinforces the need for strategy integration for the international market. Interestingly, findings from our integration hypotheses support many years of conceptual proposition in international business literature (Baron 1995; Bonardi 2004; Schuler et al. 2002; Shaffer and Hillman 2000; Wei et al. 2015a, b, c) and provide insights and enhanced understanding to researchers in the field of sustainable competitive market advantage and firm internationalization.

Similarly, environmental uncertainty provides mixed reactions. First, it shows support for the work of Zhang et al. (2020) who found that uncertainty in the market environment affects firm innovative decisions and differentiation strategies thereby improving firm performance. Second, we found that environmental uncertainty has a direct and indirect negative relationship with nonmarket strategies on customer satisfaction. This, however, negates the findings of Köseoglu et al. (2020) and depicts self-interest as the ultimate motive of organizations engaging in nonmarket strategies especially during market turbulence or uncertain market contexts to achieve sustainable competitive advantage and outwit competitor’s market strategies (Wood and Frynas 2006; Harrison and Wicks 2013). Our study is unique given its response to the long-term call to statistically address the relationship between strategy integration and firm sustainable market performance in a developed market. It also becomes the first to enhance MNEs manager’s understanding of the role of environmental uncertainty and firm capabilities and its impact on market, nonmarket, and integrated actions of the organization and how the organization needs to deploy, interact and configure internal and external capability to achieve sustainable internationalization performance. The study is also very unique owing to the fact that its results can be applied to other European countries that have a highly regulated and standardized business practices through the fostering of a cordial interrelationship between market and nonmarket orientation that serve to favor both the host-home government, consumers, and shareholders.
